# Emerging Anti-Aging Strategies - Scientific Basis and Efficacy

**DOI:** 10.14336/AD.2018.1026

**Published:** 2018-12-04

**Authors:** Ashok K. Shetty, Maheedhar Kodali, Raghavendra Upadhya, Leelavathi N. Madhu

**Affiliations:** ^1^Institute for Regenerative Medicine, Department of Molecular and Cellular Medicine, Texas A&M University Health Science Center College of Medicine, College Station, Texas 77843, USA; ^2^Olin E. Teague Veterans’ Medical Center, Central Texas Veterans Health Care System, Temple, Texas 76504, USA

**Keywords:** Aging, antioxidants, astragalus, autophagy, curcumin, intermittent fasting, neurogenesis, plasma transfusion, physical exercise, resveratrol, senescent cells, senolytics, stem cells, stem cell therapy, telomeres

## Abstract

The prevalence of age-related diseases is in an upward trend due to increased life expectancy in humans. Age-related conditions are among the leading causes of morbidity and death worldwide currently. Therefore, there is an urgent need to find apt interventions that slow down aging and reduce or postpone the incidence of debilitating age-related diseases. This review discusses the efficacy of emerging anti-aging approaches for maintaining better health in old age. There are many anti-aging strategies in development, which include procedures such as augmentation of autophagy, elimination of senescent cells, transfusion of plasma from young blood, intermittent fasting, enhancement of adult neurogenesis, physical exercise, antioxidant intake, and stem cell therapy. Multiple pre-clinical studies suggest that administration of autophagy enhancers, senolytic drugs, plasma from young blood, drugs that enhance neurogenesis and BDNF are promising approaches to sustain normal health during aging and also to postpone age-related neurodegenerative diseases such as Alzheimer’s disease. Stem cell therapy has also shown promise for improving regeneration and function of the aged or Alzheimer’s disease brain. Several of these approaches are awaiting critical appraisal in clinical trials to determine their long-term efficacy and possible adverse effects. On the other hand, procedures such as intermittent fasting, physical exercise, intake of antioxidants such as resveratrol and curcumin have shown considerable promise for improving function in aging, some of which are ready for large-scale clinical trials, as they are non-invasive, and seem to have minimal side effects. In summary, several approaches are at the forefront of becoming mainstream therapies for combating aging and postponing age-related diseases in the coming years.

The life expectancy of humans has almost doubled in developed countries in the past century. However, life extension experienced by populations resulted in an enhanced prevalence of age-related diseases [1]. Age-related ailments such as cardiovascular disease, cancer, and neurodegenerative diseases are the leading causes of morbidity and death worldwide [2-8]. A gradual process of aging in cells, tissues, and organs accompanied by the steady waning of function are normal incidents in the lifespan of an organism. When age-related changes occur gradually, individuals achieve what is known as “successful aging,” which is a condition where older individuals in sixties, seventies, and eighties show no significant disease or disability, sustain very good or reasonable cognitive function and actively participate with the life. Extending the lifespan of organisms is one of the primary goals of the aging research. However, recently, a greater emphasis has been placed on maintaining a better physical and mental health during aging because a considerable percentage of older individuals are developing a variety of age-related diseases, which is not only affecting the quality of their life but also the family members who care for them [9].

Finding ways to prevent age-related diseases is all the more important because the aging population is snowballing in the world as a result of better nutrition, effective antibiotics against infectious diseases and improved healthcare. For example, in the United States, the number of people over 65 years of age is projected to reach 98 million by 2060 from the current 46 million, which would make the percentage of people over 65 years of age to ~25% among the total population. Therefore, development of interventions that slow down the rate of aging and reduce or postpone the incidence of debilitating age-related diseases would be of immense value to improve the quality of life as well as to reduce medical costs [10, 11]. Studies in animal models have demonstrated that a variety of genetic, dietary and pharmacological interventions enhance lifespan [12-14]. Some of the anti-aging strategies that extend lifespan may also be useful for delaying the onset of age-related diseases. This review confers the efficacy of emerging anti-aging approaches for maintaining better health in old age. Approaches that have lately received significant interest will be discussed. These include strategies such as enhancement of autophagy, elimination of senescent cells using senolytics, transfusion of young blood, intermittent fasting, adult neurogenesis boost, physical exercise, antioxidant and herbal intake, and stem cell therapy.

**Table 1:** Autophagy Enhancers

Drug	Mode of action	References
Rapamycin	Inhibits mTORC1 activity	[202 - 204]
MSL (4-(4-fluorophenyl) sulfonyl-5-methylthio-2-phenyloxazole)	Increases LC3-I to LC3-II conversion without mTOR inhibition	[205, 206]
Metformin	Inhibits mTOR signaling and protein synthesis, activates adenosine monophosphate-activated protein kinase (AMPK)	[202, 207, 208]
Resveratrol	Inhibits mTOR through ATP competition, promotes LC3II and beclin-1 expression	[202, 209, 210]
Perifosine	Inhibits Akt and mTOR axis components	[211]
RSVA314 and RSVA405 (Structural similarities of Resveratrol)	Activates AMPK to inhibit mTOR and promote autophagy to increase Aβ degradation	[212]
PREP inhibitor (KYP-2047)	Targets beclin-1 and increases LC3BII and clears α-synuclein	[19, 213]
BRD5631	Autophagy through mTOR-independent pathway, NPC1, IL-1β Suppression	[214]
PP242	ATP-competitive mTOR (mTORC1 and mTORC2) inhibition	[215, 216]
Torin1	ATP-competitive mTOR (mTORC1 and mTORC2) inhibition	[217, 218]
PI103	Dual ATP-competitive mTOR (mTORC1 and mTORC2) and selective PI3KC1a inhibitor	[219]
Xestospongin B	Inhibits IP3-mediated Ca^2+^ signaling, Inhibits Beclin-1 interaction with IP3R-Bcl-2 complex	[220, 221]
Everolimus	Inhibits mTOR	[222, 223]
Spermidine	Inhibits caspase 3-mediated Beclin 1 cleavage, Inhibits acetyltransferase EP300	[224, 225]
Rottlerin	Increased LC3-II	[226, 227]
Niclosamide	Inhibits mTORC1 pathway	[228, 229]
Pifithrin-α	p53 inhibitor	[230]
Valproic acid	Suppresses Akt/mTOR pathway, Inhibits Oxidative stress	[231, 232]
IR-58	Inhibits mitochondrial membrane 44 (TIM44)-superoxide dismutase 2 (SOD2) pathway	[233]
Prazosin	Increases p-p53 and p-AMPK and decreases Akt/mTOR	[234]
Verapamil	Blocks calcium channels	[235, 236]

## Autophagy enhancement for facilitating successful aging

Autophagy is a lysosomal disintegration process, which is protective housekeeping machinery to get rid of damaged cell organelles, longstanding misfolded proteins and colonizing pathogens [15, 16]. Autophagy shields cells against stress through an activity that catabolizes intracellular elements to preserve energy homeostasis [17]. Autophagy plays a significant role in development and disease. Particularly, enhancement of autophagy has been suggested to be useful for treating a variety of conditions, including metabolic disorders, neurodegenerative diseases, cancers and infectious diseases [15]. Dysfunctional autophagy also contributes to neurodegeneration [18]. An intact autophagy function in neurons has a neuroprotective effect because autophagy facilitates the elimination of pathogenic forms of proteins such as α-synuclein involved in Parkinson’s disease [19] or tau in Alzheimer disease [20, 21]. In animal prototypes of Parkinson’s disease, treatment with autophagy enhancers such as rapamycin and lithium engendered various favorable effects, which include diminished aggregation of α-synuclein, oxidative stress, mitochondrial dysfunction, and neurodegeneration [19].

Autophagy has a significant role in the modulation of the aging process. The function of autophagy in aging is apparent from numerous studies using yeasts, worms, flies, and mice that elevated expression of autophagy-related genes is a prerequisite for lifespan extension [17, 22]. Some studies have also shown that tissue-specific expression of single autophagy gene is adequate for extending lifespan [23] whereas other studies have pointed out that distinct types of autophagy are critical for longevity as they specifically target dysfunctional cellular components and prevent their aberrant accumulation [24]. Interestingly, slow-down of aging and longevity increase achieved through food deprivation and calorie restriction (CR) are facilitated through upregulation of autophagy because dampening autophagy diminishes the anti-aging outcomes of CR [15, 25, 26]. Besides, other studies have shown that enhancing autophagy could reinstate the regenerative capacity of aging stem cells [27, 28]. Table 1 shows a list of autophagy enhancers tested in animal models and/or cell culture systems.

Thus, autophagy enhancing interventions that commence in middle age would likely facilitate successful aging and increased longevity. However, it is debatable whether the enhancement of autophagy alone is sufficient to combat aging. Besides, the selectivity of autophagy enhancers is essential as some proteins involved in the upstream autophagy-lysosomal pathway are also implicated in signaling pathways other than autophagy. Consequently, a wide-ranging stimulation of autophagy may result in dose-dependent side-effects, which makes them unsuitable for clinical translation [19]. TFrom this persecgive, targeting specific types and phases of the autophagic process is likely to have beneficial and disease-modifying effects in aging and neurodegenerative disorders. For example, agents that selectively affect core autophagy-lysosomal pathway components, including transcription factor EB, lysosomes, β-glucocerebrosidase (GCase) as well as chaperone-mediated autophagy regulators, have resulted in more precise effects on molecular pathologic processes causing Parkinson’s disease [19]. In this context, fasting and CR appear to be most beneficial and safer alternatives as they can stimulate autophagy without adverse side effects. Some dietary supplements also stimulate autophagy with other beneficial effects on the cardiovascular and nervous systems, which include resveratrol [29-32], curcumin [27], and spermidine [33-36].

### Senescent cell elimination as an anti-aging therapy

Amongst the perpetrators of organismal aging, the function of senescent cells (SCs) has caught significant interest. Cellular senescence is typified by an irreversible cell-cycle arrest, a resistance to apoptosis, and acquisition of a pro-inflammatory, tissue-destructive senescence-associated secretory phenotype, which can be engendered via different intracellular and extracellular influences [37-39]. For instance, a microbial infection can also stimulate cellular senescence [40]. Because of their incapability to proliferate, senescent cells cannot participate in standard tissue preservation and tissue repair. Rather, senescent cells disrupt the milieu by producing a plethora of bioactive factors that cause inflammation and impede regeneration [41]. Senescent cells exhibiting the cell cycle inhibitory protein p16INK4A actively propel spontaneously ensuing age-related tissue deterioration and thereby promote several diseases associated with aging [42]. Indeed, patients with neurodegenerative diseases display various markers of senescence [38, 42]. The senescence-linked secretory phenotype can also promote metabolic dysregulation, stem cell dysfunction, and loss of resilience [38]. Thus, it may be possible to defer, avert, or ameliorate multiple senescence-associated conditions just by delaying the accumulation of senescent cells or lessening the burden of senescent cells.

In several tissues and organs, senescence is a common feature during the aging process with an age-related increase in the number of senescent cells. The secretory phenotype of senescent cells fuels the chronic, pro-inflammatory systemic state known as inflammaging. Inflammaging is one of the conspicuous features of aging weakening the regenerative competence of stem cells and escalating the chance for developing age-related diseases [1]. Furthermore, a study by Musi and colleagues has shown that tau protein aggregation is accompanied by cellular senescence in the brain [43]. Additional characterization validated that senolytic drug treatment to tau transgenic mice with late-stage pathology resulted in reduced neurofibrillary tangle density, neuron loss, and ventricular enlargement. These results suggest a link between the presence of neurofibrillary tangles and cellular senescence in the brain. Another study suggested a causal connection between the accrual of senescent cells and cognition-associated neurodegeneration [37]. This study employed MAPTP301SPS19 mouse model of tau-dependent neurodegenerative disease that accumulates p16INK4A-positive senescent astrocytes and microglia. Clearance of senescent astrocytes and microglia using genetic approaches in these mice prevented gliosis, hyperphosphorylation of both soluble and insoluble tau, and degeneration of neurons in the cerebral cortex and the hippocampus [37]. Remarkably, these changes mediated by the clearance of senescent cells resulted in the rescue of cognitive function. Moreover, intervention with a senolytic drug also modulated tau aggregation [37]. Overall, this study suggested that elimination of senescent cells through administration of senolytic drugs is an efficient therapeutic avenue for treating neuro-degenerative disorders.

From the above, it appears that, the elimination of senescent cells using drugs referred to as senolytics would slow down aging and maintain better function during old age. In mice, senotherapy proved to be effective in models of accelerated aging and also during normal chronological aging. Senotherapy prolonged lifespan, rejuvenated the function of bone marrow, muscle and skin progenitor cells, improved vasomotor function and slowed down atherosclerosis progression [41, 44-46]. Anti-senescence activity has been identified in a variety of molecules, including synthetic senolytic drugs and natural compounds in certain types of food. An array of senolytics have recently received widespread interest. Several senolytic agents have been shown to alleviate multiple senescence-related phenotypes in pre-clinical models [45]. Table 2 shows a list of senolytics that have been shown to reduce senescent cells in animal models. However, their safety and reliability as anti-senescence drugs are yet to be ascertained in human clinical trials [1]. Because senescent cells spread inflammation at the systemic level through pro-oxidant and pro-inflammatory signals, foods rich in polyphenols, which exert antioxidant and antiinflammatory effects, have the promise to be utilized as "anti-senescence foods" in a nutraceutical approach to healthier aging [1].

**Table 2:** Senolytic Drugs.

Drug	Mode of action	References
Navitoclax	Targeting Bcl-2 family	[237]
Fisetin	Targeting Bcl-2 family, regulation of PI3K/AKT/NF-κβ to promote caspase-3 and inactivates ERK1/2	[238-240]
Quercetin	Targeting PI3K, Induction of HIF-1α	[241, 242]
Piperlongumine	Inhibits Akt/mTOR signaling, depletion of the androgen receptor	[243-245]
Panobinostat	Decreases Bcl-xL expression and increases acetylation of Histone 3	[246]
Geldanamycin	Inhibits HSP90	[247]
Dasatinib	Interacts with P53 and inhibits PAI-2	[241]
A1331852	Inhibits Bcl-xL	[239]
A1155463	Inhibits Bcl-xL	[239]

### Transfusion of plasma from young individuals to promote successful aging

A new procedure for limiting or reversing aspects of aging in various organs throughout the body is the transfusion of blood from the young to the aged, as molecules circulating in the young blood can rejuvenate the aging cells and tissues [47-49]. Since prehistoric periods, it has been believed that transfusion or consuming young blood or elements of young blood may rejuvenate the aging tissues and individuals [50]. A procedure called parabiosis, studied in animal models, has revived the perception of young blood as an efficient drug for slowing down aging and treating age-related diseases. Parabiosis meaning living beside is a procedure that anatomically joins two animals so that they share each other’s circulating blood. Many studies have suggested that heterochronic parabiosis, which is joining an aged animal to a young animal, leads to a multitude of beneficial anti-aging effects [51-53]. Factors in the young blood activated molecular signaling pathways in stem cells located in the liver, muscle, and brain of the old parabiont, which lead to enhanced tissue regeneration [50]. For example, transfusion of blood from a young animal to an aged animal enhanced adult neurogenesis as well as synaptic plasticity in the aged brain [54]. Additional studies suggested that several soluble factors underlie the rejuvenating effects of the young blood. The growth differentiation factor 11 (GDF11) is one of the well-characterized factors in the young blood [50]. Studies using aged mice have shown that GDF11 treatment alone can reverse age-related muscle dysfunction [55] and cardiac hypertrophy [56, 57], and enhance hippocampal neurogenesis, vasculature and markers of neuronal plasticity in the hippocampus and cerebral cortex.

Another study analyzed the effects of heterochronic parabiosis of Alzheimer’s disease (AD) transgenic mice with young healthy mice [58]. Circulation of blood from young mice into AD mice did not reduce the deposition of beta-amyloid or microglial activation. However, the procedure reversed the loss of synapses and the abnormal expression of many genes involved in critical neuronal signaling pathways in the hippocampus. Furthermore, improvements in spatial working memory and associative memory were observed with repeated intravenous administration of plasma from young healthy mice to AD mice. These results provided the idea that young plasma has the potential for slowing down the progression of the AD, and clinical trials testing the effect of young plasma in patients with the AD are already underway [59, 60]. A human pilot study found that young donor plasma infusion protocols for adults with the AD are safe and feasible though statistically significant improvements in cognition are yet to be detected.

To validate the benefits of infusion of plasma collected from younger individuals for treating aged individuals or individuals with AD, careful, placebo-controlled larger clinical trials will be required. Several issues need to be validated before recommending transfusion of plasma from younger individuals as an approach to combat aging or prevent AD. These include the age-range of younger donors having plasma that mediates beneficial effects upon transfusions into older individuals, an "optimal age" at which transfusion of plasma from younger individuals into elderly individuals would yield significant cognitive enhancement, and the volume or frequency of transfusions required to achieve substantial cognitive improvement without adverse side-effects [54]. Also, the possible side effects need to be considered, which may include acute lung injury, circulatory overload, and allergic reactions, transmission of infections, febrile nonhemolytic transfusion reactions, red blood cell alloimmunization, and hemolytic transfusion reactions [59]. There are also ethical challenges to resolve regarding the application of young blood transfusion [61].

### Intermittent fasting as a means to combat aging

Diet and eating schemes have a significant influence on the pathogenesis of many age-associated diseases, which can be gleaned from both epidemiological and experimental studies [62-66]. For example, one of the most efficient nongenetic dietary interventions that can enhance longevity is CR, involving reduced calorie intake by about 20-40% below *ad libitum* [67, 68]. A study on the dietary intake of adults living in Okinawa, a Japanese island having nearly five times higher number of centenarians than any other part of the world, revealed that Okinawan’s consumed 17% fewer calories than the average adult in Japan and 40% less than the average adult in the United States [69, 70]. Many investigations in animal prototypes have demonstrated that reduced food intake leads to increased lifespan [71]. The other beneficial effects include improved gut microbiota composition and metabolome [72], reduced age-related methylation changes in the brain [73], increased adult neurogenesis [74, 75], improved cognitive function [76-78], protection against age-related neurodegenerative disorders such as dementia [65, 79].

Recently, many studies have shown that intermittent fasting (IF) can have similar effects as CR [62, 64, 80, 81]. Benefits related to cardiovascular health include protection of heart against ischemic injury [82], reduced body mass index and blood lipids [83] improved glucose tolerance [84], and lower incidence of coronary artery disease [85]. The positive effects of IF on brain health in pre-clinical studies comprised improved cognitive function with reduced oxidative stress during middle age when IF was commenced in young adult age [86] and delayed occurrence of age-related brain impairments [87]. Moreover, in models of ischemic stroke, IF modulated forebrain neurogenesis, autophagy, and apoptosis, attenuated inflammasome activity, suppressed detrimental genetic pathways, and improved recovery [88-90]. IF was also found to be efficient for reducing neuroinflammation and preserving cognitive function after lipopolysaccharide administration, an animal model of systemic bacterial infection [91, 92]. Furthermore, IF ameliorated behavioral deficits in 3xTgAD mice [93]. In the APP/PS1 model of AD mice, IF increased β-hydroxybutyrate, which in turn, inhibited the increase of brain-derived lipoprotein lipase through down-regulation of microRNA-29a [94].

A recent study showed that IF improves tissue function and the overall health during aging in fruit flies [95]. This study demonstrated that just one month of a 2-day fed:5-day fasted IF regime at the beginning of adulthood is adequate to extend lifespan. Additional analysis revealed that IF enhanced resistance to starvation, oxidative and xenobiotic stress with a higher lipid content, which is one of the mechanisms underlying increased longevity. Furthermore, guts of flies that underwent IF displayed a significant reduction in age-related pathologies and improved gut barrier function with reduced relative bacterial abundance. Also, IF promoted beneficial effects without involving the target of rapamycin (TOR) pathway. Another study showed that 24-hour fasting enhanced intestinal stem cell function in young and aged mice by inducing a fatty acid oxidation program [96], which was evident from findings that acute genetic disruption of Cpt1a, the rate-limiting enzyme in fatty acid oxidation, abrogated the positive effect of fasting on stem cells.

How do the various beneficial effects of IF seen in animal studies relate to humans? Many variations of IF exist, the most common plans are defined here. A protocol involving fasting every day for 14-16 hours by restricting daily eating window to 8-10 hours is called the 16/8 method. A diet comprising a cycle of standard eating for five consecutive days followed by a restricted calorie intake (500-600 calories/day) in the next two days is named 5:2 plan. Another eating protocol involving a 24-hour fast, once or twice per week is termed as eat-stop diet. The other schemes of the diet include alternate day fasting and the warrior diet. The warrior diet involves starvation during the day and eating a huge meal (feasting) at night. However, it is currently unclear which one among these diverse types of IF promotes maximal benefits or any adverse side-effects on health in the long-term, though 16/8 seems to be the most popular, as it is a natural regimen to adapt.

In human studies, protocols and interpretations of IF-mediated weight loss trend varied considerably [81]. Most human IF studies did not result in significant weight loss or considerable improvements in metabolic biomarkers. Quite a few questions remain to be dealt with regarding the benefits of IF on human health. First, it is unknown whether or not IF is beneficial to commence at all ages or effective in only certain age groups. Second, the benefits of IF for improving cognitive function need to be assessed in both healthy and obese males and females of different ages with various regimens and durations of IF. For example, a short-term (28 days) IF regimen did not show significant effects on body composition, glucose metabolism or cognitive function in healthy lean men [97]. Third, examining the adverse effects of IF, if any, particularly in individuals with pre-existing health conditions will be important. Fourth, the type of dietary composition which provides maximal benefits with IF needs to be identified. Finally, it will be helpful to examine the potential positive or negative interactions between IF and physical exercise.

### Promise of neurogenesis enhancement for successful aging and preventing AD

The highest risk factor for developing sporadic late-onset forms of the AD is aging [98]. The hippocampus is one of the brain regions most severely affected in the AD, which is also a region in the brain containing neural stem cell (NSC) niches that add new neurons to the hippocampal circuitry throughout life. Within NSC niches, a subclass of slowly dividing cells expressing markers such as glial fibrillary acidic protein (GFAP), sex determining region Y-box 2 (Sox-2), brain lipid-binding protein, nestin, vimentin, and Musashi-1 are the NSCs or Type 1 cells [99-102]. When these cells divide, they display asymmetric division to maintain self-renewal as well as generate transit amplifying cells or type 2 cells. Type 2 cells express Sox-2 and Mash1 but not GFAP. They occur in clusters and proliferate actively to produce a pool of doublecortin (DCX) expressing neuroblasts or Type 3 cells and some glia [99, 103]. The neuroblasts differentiate into mature dentate granule cells expressing neuron-specific nuclear antigen (NeuN), Prox-1 and calbindin. Newly born granule cells establish afferent connectivity with the axons coming from the entorhinal cortex and efferent connectivity with the CA3 pyramidal neurons. Although the extent of hippocampal neurogenesis in adult humans has been challenged recently [104], there is enough credible evidence to support the existence of neurogenesis in the adult and aged human hippocampus. These are evident from both 5’-bromodeoxyuridine labeling studies in cancer patients [105], large-scale carbon-dating studies [106], and postmortem hippocampal tissue studies from healthy men and women with age ranging from 14-79 years [107].

Studies in animal models have shown that hippocampal neurogenesis decreases during aging [100, 108-113], and the overall decrease is exacerbated in the AD. The precise mechanistic causes underlying age-related decline in neurogenesis are unclear. Persistence of NSCs with increased quiescence and multiple changes in the microenvironment of NSC niches have been observed in rat models [100, 108, 114-117]. However, no decline was seen in the expression of multiple genes essential for NSC proliferation and neurogenesis in the dentate gyrus [118]. Overall, it appears that age-related reductions in stem cell mitogenic factors, microvasculature and cerebral blood flow, and low-grade inflammation influence reduced neurogenesis in aging because increased neurogenesis could be obtained through interventional strategies that upregulate the concentration of NSC mitogenic factors [110, 119-121] or improve the microvasculature density and diminish inflammation [30]. A recent study investigated the effect of age on NSCs and neurogenesis in the senescence-accelerated mouse prone 8 (SAMP8) strain, which is a non-transgenic short-lived strain that spontaneously develops a pathological profile similar to that of the AD [98]. SAMP8 mice displayed an accelerated loss of the NSCs that coincided with an enhanced canonical bone morphogenetic protein (BMP) signaling and increased astroglial differentiation. Remarkably, blocking the dysregulation of the BMP pathway and its pro-gliogenic effect in vivo by intracranial delivery of the antagonist Noggin restored hippocampal NSC numbers, neurogenesis, and behavior in SAMP8 mice. These results re-enforce the thought that modulation of the local microenvironment of the NSCs counteracts hippocampal dysfunction in pathological aging.

Many studies had suggested earlier that normalization of the activity of NSCs and neurogenesis slows down the evolution of AD when such interventions were applied at the early stage of the disease. The successful stratagems were physical exercise or exposure to the enriched environment [122-125], inhibition of microglial activation [126], choline supplementation [127], curcumin treatment [128] or directed expression of Neurod1 in cycling hippocampal progenitors [129]. An elegant study published recently, in addition, demonstrated that hippocampal neurogenesis is impaired before the onset of AD pathology [130]. Importantly, this investigation revealed that voluntary physical exercise could provide considerable cognitive benefit in 5×FAD mice, through enhancement of both hippocampal neurogenesis and brain-derived neurotrophic factor (BDNF). Interestingly, neither stimulation of hippocampal neurogenesis alone, nor exercise, in the absence of increased hippocampal neurogenesis, improved cognitive function. When the beneficial effects of exercise on hippocampal neurogenesis and BDNF augmentation were mimicked through genetic or pharmacological means, a similar cognitive improvement was seen in AD mice. Furthermore, suppression of hippocampal neurogenesis resulted in worsened cognitive performance and loss of preexisting dentate granule cells. Thus, pharmacological mimetics of exercise capable of enhancing both hippocampal neurogenesis and BDNF appear to be useful for improving cognitive function in the early stages of the AD [130]. In summary, combined neurogenesis and BDNF boost during adulthood and middle age may postpone aging, and prevent or delay the onset of the AD. In this context, regular or intermittent physical exercise throughout life seems to be the best and an inexpensive and non-invasive approach for continually maintaining higher levels of hippocampal neurogenesis and BDNF to stave off the AD. Pharmacological interventions mimicking physical exercise may also be useful but need rigorous testing in clinical trials for any adverse side effects.

### Physical Exercise for Modulating Aging and Preventing Dementia

The benefits of regular physical exercise (PE) for conserving the function of the cardiovascular, musculoskeletal and nervous systems are well known [131-135]. PE boosts blood flow to the working skeletal muscles by up to 100-fold and moderately to the brain; whereas organs such as liver, kidney, and testes encounter diminished blood flow during PE [136]. Even with the variability in blood flow to different organs, all organs gain from regular PE. PE attenuates the age-associated waning of maximal oxygen uptake (VO_2_max), the production of reactive oxygen species (ROS) and functional weakening of various organs [137]. Moreover, PE curbs the age-related deterioration of the cellular housekeeping system such as the proteasome, autophagy, mitophagy, and DNA repair systems, which positively impact multiple organ functions [137]. PE also leads to increased mitochondrial levels in the brain, heart, liver, and kidney [138, 139]. Thus, the PE-induced surge of maximal oxygen uptake aids organ functions including the effectiveness of antioxidant and repair systems and cellular housekeeping activities.

PE enhances immunity and modulates age-related immunosenescence. Immunosenescence is typified by the weakening of the immune system with alterations to innate and adaptive immunity [140-143]. Age-related changes affect the phenotype and function of immune cells, which can interfere with chemotaxis, intracellular killing, and the response of immune cells to pathogens. Immunosenescence contributes to the development of several age-related diseases including cardiovascular disease, AD, and diabetes in older individuals, and increases the risk for developing autoimmune diseases and chronic infection [142]. Thus, PE can also prevent age-related diseases through tempering immunosenescence. The effects of PE may also be useful for normalizing immune system function in conditions such as multiple sclerosis [144].

PE profoundly promotes the brain function in animals and humans. The aging-related cognitive decline epitomizes a critical risk factor for the onset of dementia and is linked with global neurophysiological alterations [145]. Remarkably, PE delays the age-related cognitive decline. In animal models, PE has been shown to enhance memory and mood function, accompanied with increased neurogenesis, upregulation of multiple neurotrophic factors including BDNF and improved synaptic plasticity in the hippocampus [146-152]. PE also augments neurogenesis and BDNF and prevents cognitive dysfunction in AD mice [130]. Besides, PE results in secretion of multiple factors from peripheral organs such as skeletal muscle, liver and adipose tissue, which can also influence neuronal function. A recent study demonstrated that proteins secreted by skeletal muscle cells could upregulate the expression of DCX and beta-III tubulin (TuJ-1), markers of immature neurons [153], implying that factors secreted by skeletal muscle in response to PE contribute to PE-induced increased neurogenesis.

Thus, regular PE commencing from young or middle age appears to be a necessary lifestyle change for maintaining good health in old age. Since drugs that significantly prevent age-related cognitive decline are yet to be discovered, it is vital to start PE regimen early in life when the neural reserve is still adequate, to completely avoid or at least postpone the cognitive decline. However, the amount of PE required in young or middle age to maintain healthy cognitive function in old age is yet to be ascertained. Some previous clinical trials of PE showed positive effects on cognitive performance whereas other trials showed minimal or no positive impact. Nonetheless, it is believed that PE programs that are structured, individualized, and continue for longer durations preserve cognitive performance in older adults [154, 155]. Indeed, a recent clinical trial suggested that exercising for at least 52 hours over a six months duration is associated with improved cognitive performance in older adults with or without cognitive impairment [156]. Another study showed that combined physical and cognitive activity improves or maintains cognitive and physical performance in older individuals diagnosed with mild cognitive impairment (MCI), especially the amnestic type [157]. Clinical trials on the effects of various exercises on the prevention of the AD suggested that long-term physical activity with a multicomponent cognitive intervention improves cognitive function in patients with AD [158]. Overall, the results imply that, after the onset of MCI or early AD, PE intervention alone may not be sufficient but still has the promise to improve function when combined with adequate cognitive activity.

### Promising antioxidants and herbals for promoting successful aging

Oxidative stress has a significant influence in the development of many age-related diseases, which include arthritis, diabetes, dementia, stroke, cancer, atherosclerosis, vascular diseases, obesity, osteoporosis, and metabolic syndromes [159-164]. Reactive oxygen species (ROS) are generated within the biological system to regulate cellular activities such as cell survival, stressor responses, ion channels, and inflammation [165, 166]. However, the elevation of ROS is linked to the onset and progression of aging. Particularly, ROS is believed to exacerbate the progression of age-related diseases via oxidative damage and interaction with mitochondria [167, 168]. Multiple studies imply that transient or physiological reactive oxygen species (ROS) generated by nicotinamide adenine dinucleotide phosphate (NADPH) oxidases operate as a redox signal to restore cellular homeostasis [169]. Typically, the competence to recreate cellular homeostasis gradually wanes during aging. However, long-lived animals display an ability to re-establish cellular homeostasis, which promotes healthy aging. Therefore, strategies that control NADPH oxidase activity for local and physiological redox signaling are likely to be useful for promoting healthy aging [169].

Older adults are more susceptible to oxidative stress due to a reduction in the efficiency of their endogenous antioxidant systems [170]. The heart and brain, with restricted cell renewal rate and excessive amounts of oxygen intake, are especially susceptible to this phenomenon. Typically, an inverse relationship has been observed between plasma antioxidant levels and the onset and progression of several diseases, which include cardiovascular disease [171], diabetes [172], and neurological disorders [173]. Emerging research suggests that natural antioxidants can control the autoxidation by interrupting the propagation of free radicals or by inhibiting the formation of free radicals. Through such actions, antioxidants reduce oxidative stress, improve immune function, and increase healthy longevity [164]. Antioxidants are capable of scavenging the species that initiate the peroxidation, breaking the autoxidative chain reaction, quenching free radicals, and preventing the formation of peroxides [174]. Therefore, dietary supplementation with antioxidants has received considerable interest in combating aging. This approach is also consistent with the free radical theory of aging [175, 176] that lowering the global level of ROS in the body would retard aging, increase lifespan, and prevent and treat aging-associated diseases [170, 177]. However, clinical trials with antioxidants in the elderly population have reported inconsistent findings [170]. The most promising antioxidants currently in clinical trials for treating cognitive dysfunction in aging and AD include resveratrol (RESV) and curcumin (CUR).

Regarding RESV, a clinical trial reported that 4-months of RESV treatment in middle-aged men with metabolic syndrome increased muscle turnover, lipid metabolism, and accumulation of long-chain saturated, monounsaturated, and polyunsaturated free fatty acids, and produced beneficial alterations in gut microbiota [178]. Another clinical trial showed that incorporation of RESV to standard antihypertensive treatment is efficient for reducing blood pressure to normal levels, without the need for additional antihypertensive drugs [179]. This study also implied prevention of liver damage with RESV intake, based on lower levels of hepatic enzyme glutamate-pyruvate transaminase in the serum. Additional clinical trials have shown that RESV treatment improved memory performance allied with enhanced functional connectivity between the hippocampus and the medial prefrontal cortex in healthy overweight elderly individuals [180]. RESV has also been shown to strengthen neurovascular coupling and cognitive performance in type 2 diabetes patients [181]. Besides, in individuals with mild to a moderate AD, RESV treatment modulated amyloid β-40 levels in both plasma and cerebrospinal fluid, in comparison to the placebo-treated group [182].

CUR was tested in a randomized clinical trial comprising the healthy elderly population. In this study, improved sustained attention and working memory was observed with acute CUR administration, and enhanced mood and reduced fatigue were seen with a chronic treatment [183]. Another recent clinical trial reported that daily oral ingestion of a bioavailable and safe form of CUR improves memory performance over 18 months in middle-aged and older non-demented adults [184]. Moreover, this study suggested that daily oral CUR consumption may lead to less neuropathological accumulation in the amygdala and the hypothalamus [184]. Thus, based on the results of clinical trials performed, it appears that both RESV and CUR are safe, well-tolerated and beneficial with minimal side effects. Nonetheless, detailed, long-term, larger clinical trials are needed to fully understand the efficacy of RESV and CUR for improving cognitive function in elderly people afflicted with MCI or early stage of the AD.

Several herbs or other plant products used in Chinese medicine have also been suggested to promote health and longevity [185-191]. Among these, a Chinese herb named *Astragalus membranaceus* (Huangqi) has recently been proposed to have robust antiaging activity [186]. *Astragalus membranaceus* is a prime healing herb included in many herbal formulations in the practice of conventional Chinese medication to treat a variety of diseases. It is also marketed as a life-extending tonic for humans in China [186]. The significant components of *Astragalus membranaceus* are polysaccharides, flavonoids, and saponins. The components of *Astragalus membranaceus* has been shown to increase telomerase activity, and mediate antioxidant, anti-inflammatory, immunoregulatory, anticancer, hypolipidemic, anti-hyperglycemic, hepatoprotective, expectorant, and diuretic effects [186]. An extract of the dried root of *Astragalus membranaceus*, called TA-65, has been reported to promote considerable positive effects on the immune system. Thus, *Astragalus membranaceus* may be suitable as a dietary supplement for combating vascular aging, brain aging, and cancer. However, rigorous double-blind, placebo-controlled clinical trials will be required to validate the efficacy of this herbal medicine for combating aging or AD.

### Stem cell therapy for promoting healthy brain aging and reversing AD

The efficacy of intracerebral transplantation or peripheral injection of a variety of stem cells including mesenchymal stem cells (MSCs), NSCs or glial-restricted progenitors (GRPs) has been examined in animal models to improve the function of the aging brain. A study by Hattiangady and associates demonstrated that grafting of NSCs or GRPs into the aging hippocampus leads to the stimulation of endogenous NSCs in the subgranular zone and results in increased production of new dentate granule cells [192]. This study provided the first demonstration that grafting of GRPs or NSCs is an attractive approach for improving neurogenesis in the aging hippocampus. Consistent with these findings, another study showed that implantation of a human NSC cell line (CTX0E03) into the ventricles of aged rats is also useful for increasing neurogenesis in the hippocampus [193]. Collectively, these results suggested that NSC grafting can promote regeneration in the aged brain through stimulation of endogenous neurogenesis.

A long-term study by Shetty and Hattiangady further revealed that the aged hippocampus could support robust engraftment and differentiation of cells derived from NSC grafts [194]. An interesting finding is that grafted NSCs showed an ability to establish neurogenic niches in non-neurogenic regions of the aged hippocampus. The occurrence of new neurogenic niches was evidenced through the derivation of new, immature neurons from graft-derived cells within graft cores located in the non-neurogenic regions even at three months after grafting. Sequential labeling with different birth-dating markers further confirmed that the immature neurons found within graft cores were indeed generated from graft-derived cells in the aged hippocampus. This phenomenon is beneficial if these niches can continuously produce new neurons and glia in the grafted hippocampus, as newly generated neurons and glia are expected to improve not only the microenvironment but also the plasticity and function of the aged hippocampus. Overall, these results have significance because the potential application of NSC grafting for treatment of neurodegenerative disorders at early stages of the disease progression and age-related impairments would mostly involve aged persons as recipients. Even so, further studies are essential to ascertain the functional implications of NSC grafting into the aged hippocampus and whether NSCs from other sources such as human induced pluripotent stem cells (hiPSCs) or human embryonic stem cells (hESCs) would exhibit similar behavior. Another study by Zhang and colleagues showed that retardation of aging and lifespan extension could be achieved in middle-aged mice through implantation of hypothalamic NSCs into the aged hypothalamic nucleus exhibiting inflammatory microenvironment [195]. The grafted NSCs regulated aging through the release of exosomal miRNAs in this study.

A few studies have also examined the effects of administration of MSCs in aging models. Transplantation of adipose tissue-derived MSCs (AD-MSCs) improved locomotor activity and cognitive function in the aged animals, in parallel with the recovery of acetylcholine levels in brain tissues [196]. Grafting of AD-MSCs also restored microtubule-associated protein 2 in the host brain, enhanced trk-B expression, and concentrations of BDNF and nerve growth factor. These results suggest that grafting of human ADMSCs can restore the physical and cognitive function of aged mice. In another study, Cao and colleagues examined the effects of clinical-grade human umbilical cord-derived MSCs on cognitive aging in a mouse model of aging induced by d-galactose [197]. Mice received intraperitoneal administration of MSCs once weekly for two weeks. Three months after the administration of MSCs, the hippocampal-dependent learning and memory were found to be improved in aged mice, and the synaptic plasticity was enhanced in the CA1 area of the aged hippocampus. Moreover, MSC administration improved dendritic spine density, postsynaptic density, and neurogenesis in the hippocampus. Additional analyses showed that beneficial effects of MSC administration were mediated through activation of mitogen-activated protein kinase (MAPK)-ERK-CREB signaling pathway in the aged hippocampus. Overall, this study demonstrated that peripheral administration of umbilical cord-derived MSCs is beneficial for improving the function of the aged brain.

Stem cell therapy has been shown to mediate beneficial effects in several other age-related neurodegenerative disease models. First, a study by Blurton-Jones and associates showed that hippocampal NSC transplantation rescued the spatial learning and memory deficits in aged 3xTg-AD mice [198]. The cognitive function was improved without altering the pathology of the AD. Gain-of-function and loss-of-function studies revealed that the mechanism underlying a better cognitive function involved improved hippocampal synaptic density mediated by BDNF. These are evident from observations that recombinant BDNF mimicked the beneficial effects of NSC grafting whereas depletion of NSC-derived BDNF failed to improve cognitive function or restore hippocampal synaptic density. These results suggest that NSC grafting can ameliorate complex behavioral deficits associated with AD pathology via BDNF. In a follow-up study, the same research group showed that transplantation of research grade human NSCs is also effective for improving cognitive function in two complementary models of AD [199]. Grafted human NSCs in this study significantly increased synaptic and growth-associated markers in both 3xTg-AD and CaM/Tet-DTA mice. Again, improvements in aged 3xTg-AD mice were not associated with altered Aβ or tau pathology. It appeared that human NSC transplantation improved cognitive function by enhancing endogenous synaptogenesis. Another study showed that grafting of NSCs into the brain of 12-month-old Tg2576 mice (a model of the AD) markedly improved both cognitive function with an enhanced clearance of β-amyloid, secretion of anti-inflammatory cytokines, stimulation of endogenous neurogenesis, and synapse formation [200]. However, similar NSC grafting into 15-month-old Tg2576 mice (i.e., the age at which mice display memory loss) did not result in improved cognitive dysfunction or clearance of β-amyloid. Thus, NSC therapy in the AD seems to work when grafting is performed in the early stage of the AD (i.e., before the manifestation of memory dysfunction). In summary, grafting of NSCs, GRPs or MSCs has shown promise for improving regeneration and function of the aged or AD brain. However, clinical trials using human tissue-derived stem cells (i.e., NSCs, GRPs or MSCs derived from hiPSCs or hESCs) will be required for further advancements in this field.

In addition to stem cell grafting approach, activation of endogenous cells in some regions of the body has promise for mediating regeneration during aging. In a recent study, Chunhua Zhao and colleagues propose that activation of mesenchymal tissue system may be a key for regeneration during aging [201]. The authors propose that triple energizer, which is a name used in Chinese medicine, is the largest organ in the body consisting of all functional cells originated from the MSC system during the embryonic development. This concept elaborates the origin, function and the essence of triple-energizer from the perspective of the human organ system. The triple-energizer originates from the intraembryonic coelom of the embryo, which is the mesenchymal tissue system composed of functional cells derived from mesodermal stem cells and the microenvironment. Among these cells, Flk1+ MSCs (also expressing CD44, CD29, CD105 and CD166 but not CD34, CD31, and Lin), which is a type of post-embryonic PSCs (PEPSCs), are capable of giving rise to cells of all three germ layers. This mesenchymal tissue system, closely linked with organs and tissues, plays a vital role in tissue homeostasis and aging. Besides, authors propose that this system is responsible for the proliferation and differentiation of stem cells in the body, the regeneration, and repair of tissues and organs, immunity, and regulation of a molecular network of cell metabolism in the tissue system [201, 248-249].

### Conclusions

There are many anti-aging strategies in development. Some of which have shown considerable promise for slowing down aging or delaying the onset of age-related diseases. From multiple pre-clinical studies, it appears that upregulation of autophagy through autophagy enhancers, elimination of senescent cells using senolytics, transfusion of plasma from young blood, neurogenesis and BDNF enhancement through specific drugs are promising approaches to sustain normal health during aging and also to postpone age-related diseases such as the AD. However, these approaches will require critical assessment in clinical trials to determine their long-term efficacy and lack of adverse effects on the function of various tissues and organs. On the other hand, approaches such as different types of IF, regular PE, RESV, and CUR treatment are ready for large-scale clinical trials, as they are non-invasive, and seem to have minimal side-effects. Stem cell therapy, particularly the administration of NSCs, GRPs, and MSCs, has also shown promise for improving regeneration and function of the aged or AD brain. Nonetheless, advancement of stem cell therapy for promoting successful brain aging or reversing AD symptoms and pathology would require clinical trials for safety and efficacy using cells derived from hiPSCs or hESCs, as these sources can provide an unlimited number of specific cell types needed for cell therapy in different conditions.

### Department of Defense, Department of Veterans Affairs, and United States Government Disclaimer

The contents of this article suggest the views of authors and do not represent the views of the Department of Defense, U.S. Department of Veterans Affairs, or the United States Government.
